# Comprehensive Evaluation of a Cream With Advanced Soothing Complex for Skin Recovery Following Intense Pulsed Light Therapy

**DOI:** 10.1111/jocd.70697

**Published:** 2026-02-01

**Authors:** Jianhua Zhang, Shichao Liu, Wenjiao Guo, Na Li, Li Ye

**Affiliations:** ^1^ Guangdong Sensitive Skin Care Engineering Technology Research Center Guangzhou Guangdong China; ^2^ Simcare Biotechnology Group Co., Ltd. Guangzhou Guangdong China; ^3^ N.O.D. Topia (Hongkong) Biotechnology Co., Ltd. Hongkong China; ^4^ Dermatology Hospital Southern Medical University Guangzhou Guangdong China; ^5^ Hygiene Detection Center, School of Public Health Southern Medical University (NMPA Key Laboratory for Safety Evaluation of Cosmetics, Guangdong Provincial Key Laboratory of Tropical Disease Research) Guangzhou Guangdong China

**Keywords:** Intense pulsed light, *Schisandra sphenanthera* extract, skin barrier, *Tephrosia purpurea* seed extract, 4‐tert‐butylcyclohexanol

## Abstract

**Background:**

Intense pulsed light (IPL) therapy is widely used for skin rejuvenation but frequently induces transient skin barrier impairment, erythema, and discomfort. Post‐procedure skincare targeting barrier repair and symptom relief remains a clinical priority.

**Objective:**

To evaluate the efficacy, safety, and satisfaction of a cream containing 4‐tert‐butylcyclohexanol, *Tephrosia purpurea* seed extract, and *Schisandra sphenanthera* extract (TTS cream) in facilitating skin recovery after IPL therapy.

**Methods:**

In this randomized, double‐blind, split‐face study, 30 healthy female subjects received IPL therapy. One hemiface was randomly applied TTS cream, and the contralateral side was applied a control moisturizing toner after IPL. Objective instrument assessments were conducted at baseline (D0), post‐IPL (D0′), 30 min post‐application (D30min), and on days 3 (D3), 7 (D7), 14 (D14), and 28 (D28). Subjective assessments and satisfaction surveys were concurrently collected.

**Results:**

IPL therapy significantly increased TEWL, erythema, and skin dehydration at D0′ (*p* < 0.01). At D30min, compared to the control, TTS cream significantly improved skin hydration (∆ = 12.79 vs. 0.32 C.U., *p* < 0.01). At D28, TTS cream significantly reduced erythema index (∆ = −54.61, *p* < 0.01), erythema area (∆ = −7.02%, *p* < 0.01), and TEWL (∆ = −4.41 g/m^2^ h, *p* < 0.01). Subjective assessments confirmed TTS cream significantly alleviated stinging, burning, and redness (*p* < 0.01). Satisfaction rates reached 100% for gentleness and 96.67% for symptom relief. No adverse effects were reported throughout the study.

**Conclusions:**

These findings indicate that the TTS cream effectively enhances skin barrier recovery, reduces erythema, and relieves discomfort following IPL therapy, highlighting its potential application in the postoperative care of aesthetic medicine.

## 1 Introduction

With the advancement of technology and the improvement of people's living standards, aesthetic medicine has become a popular choice for improving skin condition and treating appearance defects [1]. Aesthetic medicine incorporates advanced treatments and care procedures, with photoelectric technologies serving as an important part and being widely used around the world [2]. These clinically validated photoelectric technologies, particularly intense pulsed light (IPL), are employed to address skin aging [3], telangiectasia [4], pigmentation [5], and unwanted hair [6]. Furthermore, IPL demonstrates high efficacy in treating disfiguring dermatological conditions, such as hypertrophic scars [7], rosacea [8], and rhinophyma [4]. However, the clinical benefits of IPL are often accompanied by transient adverse effects, necessitating effective post‐procedural management strategies.

Post‐IPL adverse effects mainly result from the treatment mechanism and thermal injury risk. IPL functions via selective photothermolysis, employing a non‐coherent, polychromatic light source spanning 400–1200 nm [2]. Through the integration of narrowband cut‐off filters and precisely modulating pulse duration and energy density, IPL therapy can be tailored to address lesions of varying types, sizes, and depths [9]. However, the energy delivery inevitably poses a risk to peri‐lesional tissues. According to the U.S. Food and Drug Administration (FDA) Manufacturer and User Experience (MAUDE) database, IPL is associated with the highest complication rate (12.3%) among the same cosmetic laser procedures, including lasers, radiofrequency, ultrasound, and so on [10]. Crucially, barrier disruption is a well‐acknowledged consequence of cosmetic laser procedures like IPL [11]. Consistent with this, a study by Xia et al. on IPL treatments observed post‐treatment erythema and increased fragility of the skin's permeation barrier, potentially allowing irritants to penetrate the skin barrier more easily and increasing skin sensitivity, and giving rise to self‐reported symptoms, including itching, burning, tightness, and stinging [12]. Consequently, targeted soothing and repair measures following IPL and other phototherapy techniques are a universal approach to mitigating post‐treatment adverse effects [13]. Based on this, it is essential to develop a prognostic measure suitable for use after IPL therapy to accelerate the recovery of damaged skin and reduce the occurrence of adverse effects.

A novel post‐IPL formulation incorporating an advanced soothing complex, comprising 4‐tert‐butylcyclohexanol, *Tephrosia purpurea* seed extract, and *Schisandra sphenanthera* extract (TTS), was specifically developed to promote skin recovery and reduce the incidence of adverse effects. The thermal energy of IPL is known to activate the transient receptor potential vanilloid 1 (TRPV1) in cutaneous sensory neurons and keratinocytes, inducing transient neurosensory responses. 4‐tert‐butylcyclohexanol, a well‐characterized TRPV1 competitive antagonist, has been extensively reported in studies to mitigate TRPV1‐mediated irritation, such as redness, stinging, burning, itching, and tightness [14]. *T. purpurea* (L.) Pers. is a herbaceous plant belonging to the Fabaceae family, which is a medicinal herb historically incorporated into Ayurveda for its validated wound‐healing property [15]. Phytochemical analyses reveal it contains abundant carbohydrates, polyphenols, and organic acids. In vitro and clinical studies indicate that *T. purpurea* seed extracts promote β‐endorphin and dopamine production in keratinocytes, while exhibiting significant antioxidant activity and effectively alleviating redness [16]. *S. sphenanthera*, a traditional Chinese herbal medicine, is rich in triterpenoid compounds and lignans. Extracts of *S. sphenanthera* exhibited potent antioxidant, anti‐inflammatory, anti‐allergic, soothing, and anti‐aging properties [17]. Therefore, the TTS cream was incorporated into the post‐IPL care protocol with the expectation that it would effectively alleviate discomfort, accelerate barrier recovery, suppress inflammation, and thereby improve overall treatment outcomes. This study aimed to clinically evaluate the effectiveness, safety, and satisfaction of the TTS cream in facilitating skin recovery after IPL therapy and elucidate the benefits of targeted care for improving IPL prognosis.

## 2 Materials and Methods

### 2.1 Methods

The randomized, double‐blind, self‐controlled trial complied with the Helsinki Declaration and the ICH GCP guidelines as applicable to cosmetic samples. All subjects were advised of the experimental risks prior to providing written informed consent and participated voluntarily. The subjects consented to the publication of associated images in this paper.

### 2.2 Subjects

A total of 30 healthy female subjects aged 24–44 years, with an average age of 35.47 ± 6.18, were enrolled in this study. Inclusion criteria comprised (a) age between 18 and 45 years and in good general health; (b) willingness to receive IPL therapy; (c) provision of voluntary written informed consent; and (d) commitment to comply with all experimental procedures. Exclusion criteria comprised (a) application of any topical medication on the test area within 2 months prior to enrollment; (b) use of antihistamines within 1 week before enrollment, or use of retinoids or immunosuppressants within 1 month before enrollment; (c) active inflammatory skin disorders (e.g., rosacea, seborrheic dermatitis, atopic dermatitis) or photosensitive skin conditions (e.g., vitiligo, photosensitivity dermatitis, lupus erythematosus); (d) pregnancy, lactation, planned pregnancy within 6 months, or being within 6 months postpartum; (e) presence of significant skin abnormalities in/near test areas that could interfere with test results; (f) participation in other facial clinical trials within 2 months prior to enrollment; (g) any other condition deemed clinically unsuitable for participation by investigators.

### 2.3 Procedure

**FIGURE 1:**
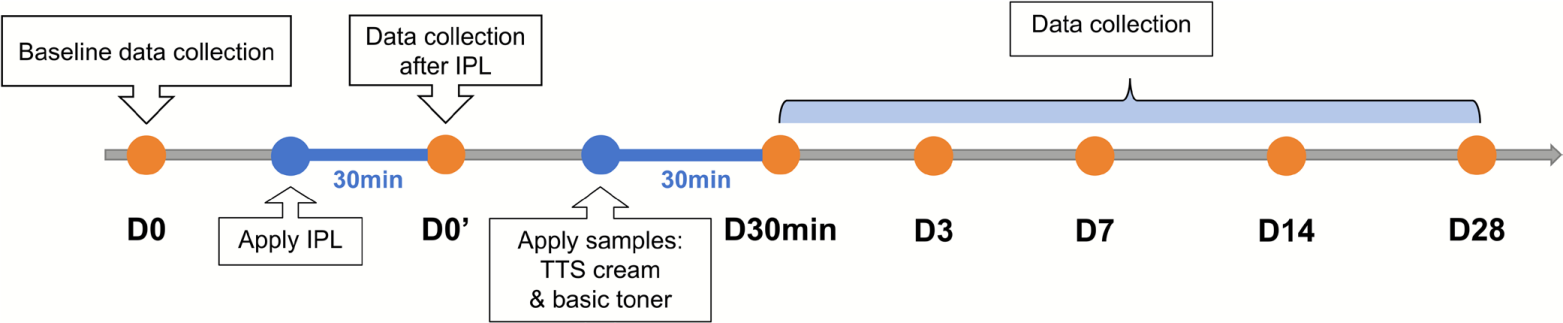
Study procedure diagram.

**FIGURE 2:**
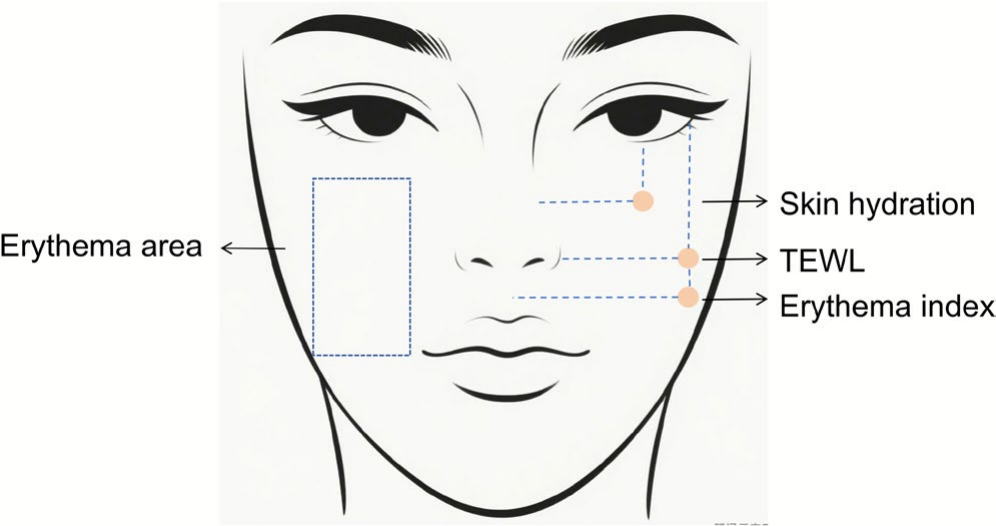
Skin parameter test location diagram.

The study procedure was shown in Figure 1. Specifically, under researcher supervision, subjects received standardized facial preparation comprising cleansing with a mild cleanser and blot drying with lint‐free wipes. Subjects subsequently acclimated for 30 min in a controlled environment (21°C ± 1°C, 50% ± 10% RH). Before IPL treatment (D0), the erythema area ratio, transepidermal water loss (TEWL), skin hydration, and erythema index were measured using the VISIA‐CR, AquaFlux AF200 evaporimeter (Biox Systems Ltd., UK), corneometer CM 825 (Courage+Khazaka Electronic GmbH, Germany), and Mexameter MX 18. The measurement locations for the subjects were shown in Figure 2. Two dermatologists independently assessed the skin condition severity of subjects using a 10‐point Visual Analog Scale (0 = absent to 9 = severe) [18], while subjects completed self‐assessment questionnaires regarding skin condition.

Non‐ablative IPL therapy was administered using the Lumenis M22 system (Lumenis Ltd., Israel) with these parameters: wavelength 540–580 nm, dual‐pulse mode (on‐Ntime 3.0 ms, delay 28 ms, off‐time 3.8 ms), and fluence 14–18 J/cm^2^. After treatment, subjects acclimated for 30 min in a controlled environment. The erythema area ratio, TEWL, skin hydration, erythema index, and subjective assessment were measured (D0′). Subsequently, the Traucr bovine collagen dressing (Guangzhou Trauer Biotechnology Co. Ltd., China) was applied to the face of subjects for targeted IPL‐injured skin barrier restoration.

Using computer‐generated randomization, the left or right hemiface was assigned to the experimental group receiving the TTS cream, with the contralateral hemiface receiving a standardized moisturizing toner as the control. The erythema area ratio, TEWL, skin hydration, erythema index, and subjective assessment were measured at 30 min after application (D30min). The 28‐day study included follow‐up visits at D3, D7, D14, and D28. During the experimental period, subjects were instructed to apply broad‐spectrum sunscreen (Curel moisturizing sunscreen lotion) to protect facial skin and limit the duration of UV exposure, aiming to minimize potential skin damage. Any adverse effects or serious adverse effects would be recorded throughout the study.

### 2.4 Sample and Treatment

The test sample in this study was a soothing and repairing cream containing 4‐tert‐butylcyclohexanol, *Tephrosia purpurea* seed extract, and *Schisandra sphenanthera* extract (TTS) on the left or right hemiface of subjects. 4‐tert‐Butylcyclohexanol was incorporated at 1% (w/w). This concentration is supported by published clinical data indicating its efficacy in reducing neurosensory discomfort through antagonism of the TRPV1 receptor [14]. *Tephrosia purpurea* seed extract and *Schisandra sphenanthera* extract were used at 0.5% (w/w), respectively. This dosage was selected based on in vitro studies (provided by the supplier) demonstrating significant anti‐inflammatory and antioxidant activity at this concentration [16]. The remaining part of the formulation consists of standard emulsifiers, emollients, humectants such as glycerin and squalane, and barrier‐supporting lipids such as ceramides. This ensures good skin compatibility and provides basic moisturization. The complete ingredient list of the test cream and the control toner is provided below for full transparency:

TTS cream: aqua, glycerin, butylene glycol, squalane, dimethicone, caprylic/capric triglyceride, pentylene glycol, 4‐tert‐butylcyclohexanol, *T. purpurea* seed extract, *S. sphenanthera* extract, 1,2‐hexanediol, hydroxyacetophenone, sodium hyaluronate, ceramide, acrylates/c10‐30 alkyl acrylate crosspolymer, and xanthan gum.

Given IPL‐induced transient skin barrier impairment, the contralateral hemiface of subjects was treated with a commercially available basic moisturizing toner application for barrier restoration. The compositions of toner were aqua, propanediol, butylene glycol, bis‐PEG‐18 methyl ether dimethyl silane, glycerin, *Centella asiatica* leaf extract, D*endrobium officinale* stem extract, sodium hyaluronate, ectoin, 1,2‐hexanediol, hydroxyacetophenone, bisabolol, pentylene glycol, ceramide NP, sodium stearoyl glutamate, tocopherol, disodium EDTA, and carbomer. Both samples were applied twice daily for 28 days.

### 2.5 Statistical Analysis

The experimental data were statistically analyzed using SPSS 24.0 software (IBM Corp., Armonk, NY, USA). The data collected was presented in mean ± standard deviation (SD) format. Paired *t*‐test or Wilcoxon signed‐rank tests were conducted to compare data between TTS cream and control. Intergroup differences were compared by one‐way analysis of variance (ANOVA), with significance levels indicated as follows: *p < 0.05 and **p < 0.01.

## 3 Results

### 3.1 Objective Instrumental Assessment

**FIGURE 3:**
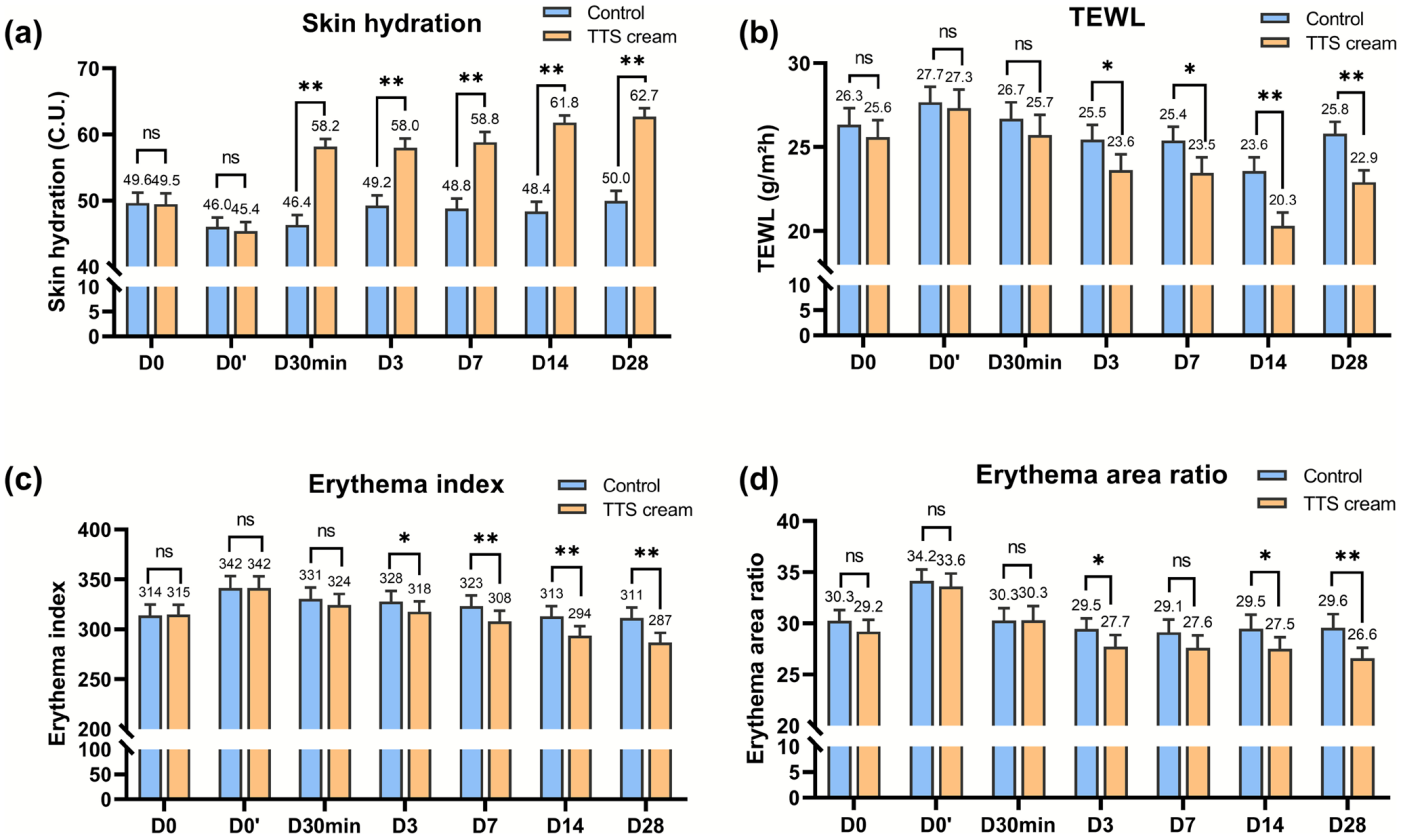
Changes in skin parameters during testing. (a) The changes of skin hydration; (b) The changes of TEWL; (c) The changes of erythema index; (d) The changes of erythema area ratio. D0: baseline; D0′: after IPL therapy; D30min: 30 min after application of the test sample; D3, D7, D14, and D28: 3, 7, 14, and 28 days after application of the test sample. *p < 0.05, **p < 0.01 vs. Control.

No significant baseline differences in erythema area, TEWL, skin hydration, or erythema index were observed between the TTS cream and control groups (Figure 3). IPL therapy induced significant alterations in skin parameters at D0′. Compared to baseline, skin hydration decreased, while transepidermal water loss (TEWL), the erythema index, and area ratio increased (both *p* < 0.01) (Table 1). After 30 min of TTS cream treatment (D30min), compared to D0′, skin hydration was increased from 45.38 ± 1.39 C.U. to 58.17 ± 1.16 C.U. (Δ = 12.79 C.U., *p* < 0.01), significantly higher than the control group (Δ = 0.32 C.U., *p* < 0.01) (Table 1, Figure 3a). TEWL, the facial erythema index, and the area ratio also showed mild improvements at D30min, but there was no significant difference compared to the control group (Figure 3b–d).

**TABLE 1:** Changes from baseline or D0′ in skin parameters at both sides during follow‐up (*n* = 30).

Skin parameters	Time points	Mean change from baseline, mean ± SD		Mean change from D0′, mean ± SD		*p* value vs. baseline (D0) or D0′	
		Control	TTS cream	Control	TTS cream	Control	TTS cream
Skin hydration	D0′	−3.61 ± 0.028	−4.11 ± 0.028			< 0.01**	< 0.01**
	D30min			0.32 ± 0.032	12.79 ± 0.026	0.454	< 0.01**
	D3			3.21 ± 0.034	12.61 ± 0.031	< 0.01**	< 0.01**
	D7			2.76 ± 0.033	13.44 ± 0.034	< 0.01**	< 0.01**
	D14			2.33 ± 0.033	16.44 ± 0.024	0.004**	< 0.01**
	D28			3.96 ± 0.032	17.35 ± 0.028	< 0.01**	< 0.01**
TEWL	D0′	1.34 ± 0.035	1.72 ± 0.043			< 0.01**	< 0.01**
	D30min			−0.98 ± 0.035	−1.6 ± 0.044	0.014*	< 0.01**
	D3			−2.22 ± 0.031	−3.69 ± 0.034	< 0.01**	< 0.01**
	D7			−2.27 ± 0.029	−3.85 ± 0.034	< 0.01**	< 0.01**
	D14			−4.09 ± 0.030	−7.01 ± 0.029	< 0.01**	< 0.01**
	D28			−1.87 ± 0.026	−4.41 ± 0.026	0.005**	< 0.01**
Erythema index	D0′	27.58 ± 0.037	26.84 ± 0.037			< 0.01**	< 0.01**
	D30min			−10.94 ± 0.034	−17.12 ± 0.032	< 0.01**	< 0.01**
	D3			−13.78 ± 0.032	−23.72 ± 0.030	< 0.01**	< 0.01**
	D7			−18.24 ± 0.031	−33.43 ± 0.031	< 0.01**	< 0.01**
	D14			−28.62 ± 0.030	−47.86 ± 0.028	< 0.01**	< 0.01**
	D28			−30.2 ± 0.031	−54.61 ± 0.028	< 0.01**	< 0.01**
Erythema area	D0′	3.89 ± 0.038	4.40 ± 0.043			< 0.01**	< 0.01**
	D30min			−3.87 ± 0.035	−3.30 ± 0.042	< 0.01**	< 0.01**
	D3			−4.69 ± 0.030	−5.88 ± 0.034	< 0.01**	< 0.01**
	D7			−5.01 ± 0.036	−5.98 ± 0.035	< 0.01**	< 0.01**
	D14			−4.66 ± 0.040	−6.07 ± 0.032	< 0.01**	< 0.01**
	D28			−4.56 ± 0.039	−7.02 ± 0.031	< 0.01**	< 0.01**

*Note:* **p < 0.01 vs. baseline (D0)/D0′.

**FIGURE 4:**
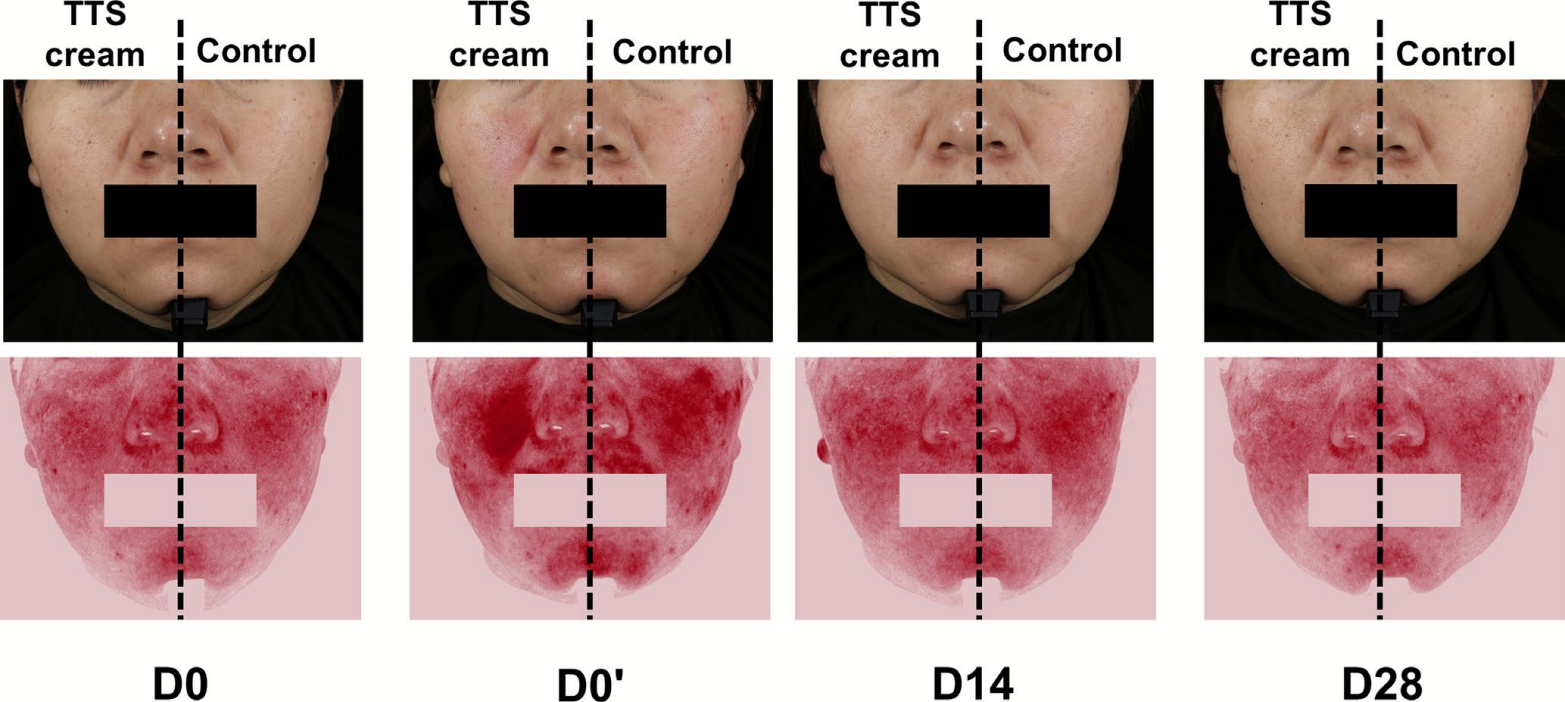
Typical images of subject no. 27 using TTS cream or control toner after IPL therapy.

During the 28day study, the TTS cream group showed greater improvement in all measured skin parameters than the control group at each evaluation time point (Table 1). Skin hydration and TEWL of the TTS cream group showed significant improvement at D3, D7, D14, and D28 compared to D0′ (skin hydration: Δ = 17.35 C.U. at D28, TEWL: Δ = −4.41 g/m^2^ h at D28, all *p* < 0.01), significant differences compared to the control group (*p* < 0.05) (Figure 3a,b). At the same time, the facial erythema index and area ratio of the TTS cream group showed significant reductions from D3 to D28 (erythema index: Δ = −54.61 at D28, erythema area ratio: Δ = −7.02% at D28, all *p* < 0.01) (Table 1). At D28, the TTS cream group exhibited significant reductions in erythema index (286.89 ± 9.59 vs. control 311.32 ± 10.73, *p* < 0.01) and area ratio (26.59% ± 1.03% vs. 29.59% ± 1.32%, *p* < 0.01), both showing greater improvement than the control group and lower than the baseline, while the control group showed no significant changes comparable to the baseline(Table 1, Figure 4).

### 3.2 Subjective Assessment

Dermatologists and subjects evaluated the efficacy of TTS cream and the control on skin using a 10‐point Visual Analog Scale (VAS, 0 = absent to 9 = severe) after IPL therapy. The results of the physician VAS score were shown in Table 2. Following IPL therapy (D0′), dermatologists' VAS scores for facial redness, dryness, and desquamation increased. Particularly, redness scores increased from 4.80 to 5.83, significantly higher than the baseline. However, after using the TTS cream, improvements in redness, dryness, and desquamation were significantly greater than those of the control at D7, D14, and D28 (*p* < 0.01) (Table 2). The VAS scores of the subjects were shown in Table 3. Following IPL therapy (D0′), the subjects' VAS scores for stinging, burning, and redness significantly increased compared to baseline (*p* < 0.01). After 30 min of TTS cream application, the subjects reported a significant reduction in discomfort such as stinging, burning, redness, itching, tightness, dryness, desquamation, and roughness, with greater improvement than the control group (all *p* < 0.01 vs. control).

**TABLE 2:** Physician‐assessed VAS score (*n* = 30).

Time points	Redness			Dryness			Desquamation		
	Control	TTS cream	*p*	Control	TTS cream	*p*	Control	TTS cream	*p*
D0	4.80	4.87	0.317	4.63	4.63	1	3.37	3.53	0.025*
D0′	5.83	5.87	0.705	4.90	4.97	0.48	3.47	3.57	0.083
D30min	5.23	5.17	0.48	4.83	4.07	< 0.01**	3.43	3.00	0.001**
D3	5.07	5.07	1	4.50	3.63	< 0.01**	3.30	2.97	0.002**
D7	4.87	4.60	0.011*	4.60	3.63	< 0.01**	3.23	2.93	0.020*
D14	4.83	4.43	0.001**	4.57	3.57	< 0.01**	3.27	2.87	0.01*
D28	4.70	4.37	0.004**	4.40	3.57	< 0.01**	3.27	2.90	0.01*

*Note:* *p < 0.05, **p < 0.01.

**TABLE 3:** Self‐assessed VAS score of subjects (*n* = 30).

	Group.	D0	D0′	D30min	D3	D7	D14	D28
Tingling	Control	0.53	2.53	1.93	1.17	0.93	0.7	0.37
	TTS cream	0.53	2.53	1	0.8	0.63	0.37	0.3
	*p* value	1	1	0.007**	0.005**	0.013*	0.004**	0.48
Burning	Control	1.03	3	2.37	1.03	1.03	0.87	0.5
	TTS cream	1.03	3	1.43	0.63	0.57	0.5	0.43
	*p* value	1	1	0.01*	0.006**	0.006**	0.031*	0.483
Redness	Control	2.57	3.7	2.97	2.13	1.8	1.7	1.47
	TTS cream	2.57	3.7	2.2	1.77	1.4	1.33	0.9
	*p* value	1	1	0.003**	0.002**	0.078	0.021*	0.01**
Itching	Control	1.07	1.33	1.33	1.13	1.07	1	0.67
	TTS cream	1.07	1.33	0.87	0.8	0.6	0.6	0.47
	*p* value	1	1	0.006**	0.004**	0.008**	0.021*	0.244
Tightness	Control	2.57	2.6	2.03	2	2.03	1.7	1.37
	TTS cream	2.57	2.6	1.13	1.1	1.13	1.03	0.73
	*p* value	1	1	0.01*	0.01*	0.01*	0.01*	0.003**
Dryness	Control	3.2	2.97	2.4	2.43	2.13	1.93	1.53
	TTS cream	3.2	2.97	1.3	1.5	1.37	1.2	0.83
	*p* value	1	1	0.01*	0.01*	0.01*	0.01*	0.004**
Desquamation	Control	1.23	1.4	1	1.03	0.97	0.83	0.43
	TTS cream	1.23	1.4	0.63	0.67	0.5	0.37	0.37
	*p* value	1	1	0.039*	0.008**	0.010*	0.008**	0.492
Roughness	Control	2.73	2.47	2.27	2.17	1.77	1.43	1.17
	TTS cream	2.73	2.47	1.47	1.6	1.3	1	0.77
	*p* value	1	1	0.01*	0.01*	0.005**	0.008**	0.041*

*Note:* *p < 0.05, **p < 0.01.

### 3.3 Subject Satisfaction Assessment and Safety

**FIGURE 5:**
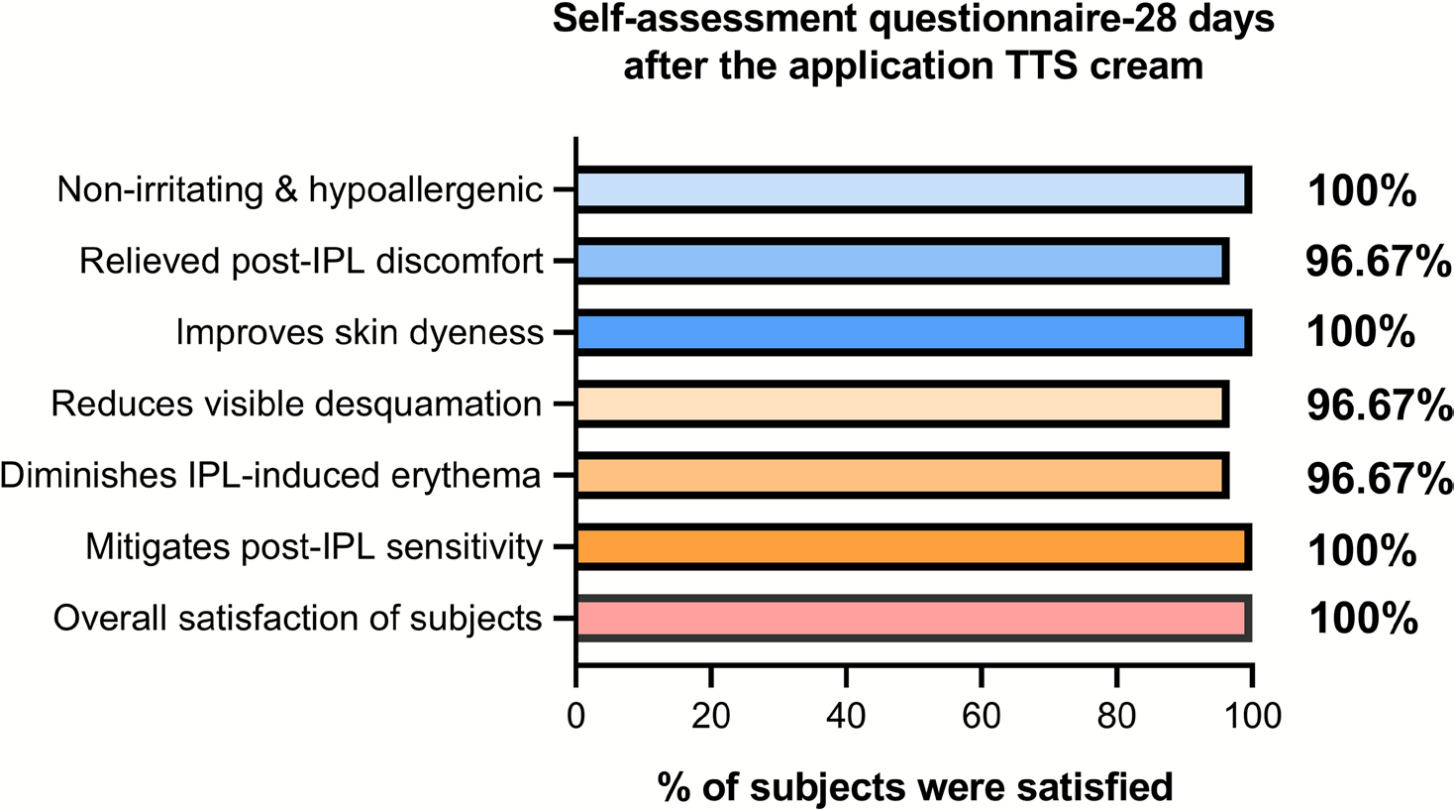
The satisfaction of the subjects with the efficacy of the TTS cream after IPL therapy.

A total of 30 subjects were randomly assigned to use either TTS cream or basic toner on one side of their face for 28 days following IPL therapy. 100% of subjects reported satisfaction with the TTS cream's gentle, non‐irritating properties when used post‐IPL (Figure 5). 96.67% of subjects reported satisfaction with the cream's ability to alleviate post‐IPL discomfort, improve facial desquamation, and reduce redness. The overall satisfaction rate for the TTS cream was 100%. No adverse reactions related to the TTS cream were observed during the testing process.

## 4 Discussion

Appearance plays an important role in social and professional settings and directly influences others' first impressions of an individual [19]. The negative psychological effects caused by physical appearance defects now constitute a clinically significant disease burden, comparable in severity to physical illnesses, as recognized by contemporary epidemiological research [20]. In this context, a range of non‐ablative aesthetic medicine techniques are utilized for appearance defect management and facial skin care. Among these, IPL therapy has emerged as the preferred modality for individuals seeking to enhance skin health and appearance, owing to its well‐documented efficacy and broad applicability [21]. However, it should not be overlooked that the patient reported skin barrier damage and immediate discomfort caused by IPL treatment. To address these issues, this study developed a post‐IPL repair cream containing 4‐tert‐butylcyclohexanol, *Tephrosia purpurea* seed extract, and *Schisandra sphenanthera* extract (TTS). This intervention not only mitigates the incidence of adverse effects but also accelerates skin recovery and enhances patient satisfaction, suggesting its potential as a standard care protocol following IPL therapy.

In this study, IPL therapy induced facial erythema, reduced skin hydration, and impaired barrier function in subjects. These effects were quantified by elevated TEWL, erythema index, and erythema area ratio (Figure 3, Table 1). These findings were consistent with the known transient side effects of IPL therapy. This is supported by clinical reports and post‐market surveillance data for at‐home IPL devices, which document mild‐to‐moderate adverse events including erythema (16.0%), pain (27.8%), and burning sensation (18.7%) [22, 23]. IPL therapy is based on the principle of selective photothermolysis, which exhibits high tissue penetrative capacity, directly targeting deep dermal layers to rapidly heat subcutaneous adipose tissue and induce localized cutaneous warming [24]. This increase in heat may be responsible for the transient rise in transepidermal water loss (TEWL) at the test site. While erythema reflects a localized inflammatory response by heat or oxidative stress and cytokine release, such as PGE_2_ and IL‐1β.

To counteract these effects, the post‐IPL topical therapeutic formulation developed in this study incorporates seed extracts from *T. purpurea* and *S. sphenanthera*, which synergistically target oxidative stress and inflammation. *T. purpurea* seed extract activates the nuclear factor erythroid 2‐related factor 2 (Nrf2) signaling pathway, up‐regulating antioxidant enzyme expression, including heme oxygenase‐1 (HMOX‐1) to enhance cellular defense against oxidative stress and mitigate heat‐induced damage [16, 25]. Concurrently, it modulates neuroimmune responses by reducing cortisol and elevating β‐endorphin and dopamine levels, thereby inhibiting proinflammatory cytokine release and promoting cutaneous homeostasis [16]. Similarly, *S. sphenanthera* extract is enriched with lignans and flavonoids, which collectively inhibit the production of pro‐inflammatory cytokines such as COX‐2 and PGE_2_ and exhibit potent free radical scavenging activity. These combined effects effectively alleviate oxidative stress‐related inflammation [17]. These mechanistic actions translate into significant clinical benefits. The formulations containing 2% *T. purpurea* seed extract mitigate facial erythema by reducing a* values and enhance skin brightness through increased L* and ITA° values [16]. *S. sphenanthera* extract enhances keratinocyte survival and metabolic activity, thus showing potential in strengthening the epidermal barrier function. Its high polysaccharide content also improves skin hydration and reduces skin water loss [17, 26]. As expected, compared to the control side using basic moisturizing toner, the TTS‐treated side after IPL therapy showed greater improvement in skin hydration, TEWL, erythema index, and erythema area ratio at 30 min post‐treatment and on days 3, 7, 14, and 28 (Figure 3). All measured facial parameters of the TTS cream improved significantly from baseline by day 28, suggesting that basic moisturizing care may not provide adequate restoration after IPL treatment and highlighting the need for targeted barrier repair formulations. The TTS cream developed in this study not only promotes recovery from IPL‐induced skin damage but may also improve the overall efficacy of IPL therapy.

On the other hand, the pathogenesis of erythema within adverse effects is mechanistically linked to IPL‐induced activation of transient receptor potential vanilloid 1 (TRPV1). The TRPV1, a non‐selective cation channel abundantly expressed in epidermal keratinocytes, is activated and sensitized by high temperatures (> 43°C), acidic environments (pH < 6), and capsaicin [27]. IPL therapy, which relies on photothermal energy transfer, induces transient peak skin temperatures exceeding the activation threshold of TRPV1 [28]. As a key mediator of neurogenic inflammatory responses, TRPV1 activation indirectly upregulates the expression of vascular endothelial growth factor (VEGF). This growth factor potentiates vascular reactivity and induces vasodilation through modulation of endothelial cell signaling pathways [29, 30]. The TTS cream was formulated with 4‐tert‐butylcyclohexanol, a potent TRPV1 inhibitor previously shown to ameliorate sensitive skin conditions [31]. Through suppression of TRPV1 activation in keratinocytes, the TTS cream suppresses neurogenic inflammation and vascular hyperreactivity, thereby reducing facial erythema.

Meanwhile, activation of TRPV1 and inflammatory responses trigger downstream signaling cascades that manifest as subjective thermal discomfort, such as burning, stinging, and itching [27, 32]. Multiple clinical trials have consistently reported mild to moderate pain in subjects receiving IPL therapy [33, 34]. In this study, this thermal insult directly contributes to post‐IPL treatment subjective symptomatology, as evidenced by significant increases in the subjects' reported stinging, redness, burning, and itching VAS scores following facial IPL (Table 3). Following IPL treatment, application of TTS cream significantly reduced discomfort; the VAS scores of stinging, burning, and itching were significantly reduced (*p* < 0.05) within 30 min, with the burning sensation showing the most pronounced reduction by 52.33% (3.00 to 1.43) (Table 3). The improvements in all VAS scores were significantly greater than those in the control group, highlighting the efficacy of targeted soothing interventions after IPL procedures. Importantly, the cream exhibited an excellent safety profile. No adverse events were reported throughout the study, even with the presence of increased facial sensitivity following IPL treatment. These findings confirm that TTS cream effectively blocks TRPV1 and inflammation‐mediated thermal nociception, while supporting its expanded use in managing IPL‐induced discomfort beyond conventional sensitive skin applications.

Compared to the control basic moisturizing toner, the TTS cream showed superior efficacy in ameliorating facial erythema, enhancing cutaneous moisture, and reducing TEWL. Furthermore, it exhibited unique advantages in lowering VAS scores assessing both subject‐reported discomfort and physician‐evaluated clinical severity. Following application of the TTS cream, the global satisfaction rate among study subjects reached 100%, and the incidence of adverse effects was eliminated. These results significantly enhanced treatment adherence and patient satisfaction with IPL therapy (Figure 5). The double‐blind, split‐face study design effectively eliminated inter‐subject variability, objectively validating the efficacy of the TTS cream. Notably, the experimental group received a cream formulated with 4‐tert‐butylcyclohexanol, *T. purpurea* seed extract, and *S. sphenanthera* extract, whereas the control group was administered a basic moisturizing toner. Consequently, the study design does not fully exclude the potential contribution of the base formulation to the observed efficacy, rather than solely attributing it to the active ingredients. To further clarify the pharmacological effects of the active components, future studies should employ an identical base formulation devoid of active ingredients as a control group. Concurrently, the absence of capsaicin stimulation assays in this study constrained the investigation of neurogenic pain pathways. Future investigations are therefore advised to quantify the desensitizing effect of TTS cream on TRPV1, which will further elucidate its role in modulating sensory signaling and pain perception. Although TTS cream has not shown any adverse effects, its effects on mature skin (aged 45 and above) have not yet been confirmed. Since IPL is often used for the management of melasma and skin aging, extending the study to a photoaging cohort would strengthen its generalizability.

## 5 Conclusion

In summary, the TTS cream formulated with the advanced soothing complex, comprising 4‐tert‐butylcyclohexanol, *Tephrosia purpurea* seed extract, and *Schisandra sphenanthera* extract, showed multidimensional efficacy. It not only inhibited inflammatory responses and promoted skin barrier repair but also attenuated IPL‐induced neurovascular hypersensitivity. Clinically, this formulation effectively addressed facial barrier dysfunction and erythema in post‐IPL patients, significantly enhancing patient‐reported subjective well‐being. Additionally, the TTS cream mitigates adverse effect risk, accelerates recovery, and improves overall patient satisfaction. This study confirms the considerable clinical benefits of TTS cream in supporting post‐IPL recovery.

## Author Contributions

Jianhua Zhang: conception and design, acquisition of data, analysis and interpretation of data, critical revision. Shichao Liu: conception and design, project administration, funding acquisition. Wenjiao Guo: writing‐review and editing, analysis, and interpretation of data. Na Li: writing‐original draft preparation and interpretation of data. Li Ye: conception and design, acquisition of data, analysis and interpretation of data, critical revision. All authors have read and agreed to the published version of the manuscript.

## Funding

The entire study was supported by Simcare Biotechnology Group Co. Ltd.

## Ethics Statement

The randomized, double‐blind, self‐controlled trial was conducted at the Dermatology Hospital of Southern Medical University (Guangzhou, China) in August 2023. This study complied with the Helsinki Declaration and the ICH GCP guidelines as applicable to cosmetic samples. The study protocol was approved by the hospital's Ethics Committee (Approval No.: 2023124).

## Consent

All subjects were advised of the experimental risks prior to providing written informed consent and participated voluntarily. The subjects consented to the publication of associated images in this paper.

## Conflicts of Interest

The authors declare no conflicts of interest.

## Data Availability

The data that support the findings of this study are available from the corresponding author upon reasonable request.
